# Relationship between oxidative balance score and risk of sleep-related problems

**DOI:** 10.3389/fnut.2025.1571971

**Published:** 2025-04-28

**Authors:** Piao Chen, Jin Wang, Ling Liu, Xiaoling Liu

**Affiliations:** Department of Tuberculosis, The Second Hospital of Nanjing, Nanjing, China

**Keywords:** oxidative stress, sleep duration, obstructive sleep apnea, diet, NHANES

## Abstract

**Background:**

Current research predominantly emphasizes the impact of diet on sleep, while overlooking the role of oxidative effects influenced by lifestyle factors. The Oxidative Balance Score (OBS) provides a comprehensive measure of individual overall oxidative stress exposure, integrating 16 dietary nutrients and 4 lifestyle factors that affect oxidative processes.

**Methods:**

To explore the relationship between OBS and sleep-related problems, data from the National Health and Nutrition Examination Survey (NHANES) conducted between 2005 and 2008 were utilized for cross-sectional analyses. OBS was calculated following previously validated methods. Sleep-related problems were assessed based on self-reported data, including sleep duration, sleep-onset latency, obstructive sleep apnea (OSA), sleep problems and day sleepiness. Weighted logistic regression was applied to estimate OR and 95% CI. To examine potential nonlinear relationships between OBS and the risk of sleep-related problems, generalized additive models and two-part linear regression models were employed. Additionally, these models were used to identify points of inflection.

**Results:**

Logistic regression analysis revealed an inverse association between OBS and the risk of insufficient sleep hours (OR = 0.98, 95% CI, 0.96 -0.99, p < 0.01). Generalized additive models and two-part linear regression models identified a nonlinear relationship between OBS and the risk of developing OSA and excessive sleep onset latency, with inflection points of 17.5 score and 10.5 score, respectively.

**Conclusion:**

Our study showed an inverse linear relationship between OBS and the risk of insufficient sleep hours, alongside a nonlinear relationship between OBS and the risks of developing OSA and excessive sleep onset latency.

## 1 Introduction

Sleep quality is a crucial determinant of an individual’s quality of life (1), as it is intricately linked to the prevalence of numerous diseases and indirectly affects economic costs through its effects on human safety and productivity (2). Research indicates that approximately 45% of adults in the United States suffer from insufficient sleep (3). Research estimates that the economic costs associated with sleep deprivation in the United States range from $288–$411 billion, accounting for 2.28% of the United States’ GDP, and this figure is projected to rise to $318–$456 billion by 2030 (3). Given these substantial costs, addressing and studying sleep-related issues is essential for both individuals and society.

Some studies have identified a direct relationship between the balance of antioxidants and pro-oxidants and sleep quality, suggesting that an appropriate daily intake of these compounds may enhance sleep (4, 5). However, most existing research primarily focuses on the effects of overall dietary patterns or specific nutrient, such as vitamin D (6), omega-3 polyunsaturated fatty acids (7), while overlooking the influence of oxidative stress related to lifestyle factors. The Oxidative Balance Score (OBS) offers a comprehensive measure of overall oxidative stress exposure within a population by calculating a total score based on an individual’s smoking habits, alcohol consumption, physical activity and dietary intake of pro-oxidants and antioxidants (8). OBS has been shown to correlate significantly with various biomarkers of oxidative stress, including 8-hydroxydeoxyguanosine (9), superoxide dismutase (10), glutathione peroxidase (10) and C-reactive protein (11). Nevertheless, most studies exploring the relationship between OBS and sleep have been limited to specific populations, and the sleep indicators involved have not been consistent (12–14).

Therefore, to fill the knowledge gap on the relationship between OBS and sleep disorders, we utilized public data from National Health and Nutrition Examination Survey (NHANES) to assess the impact of OBS on various sleep disorders, including sleep duration, sleep-onset latency, obstructive sleep apnea (OSA), sleep problems and day sleepiness.

## 2 Subjects and methods

### 2.1 Data and sample sources

The NHANES is a periodic national program conducted by the National Center for Health Statistics (NCHS) to assess the nutritional and health status of adults in the United States (15). This program employs a series of multi-stage stratified sample designs to ensure comprehensive and representative data collection. At the time of recruitment, the NCHS obtains informed consent from each participant through a standardized consent form (15). For more detailed information regarding the survey design, codebook, and methodologies employed in NHANES, interested individuals can visit the official NHANES website at https://www.cdc.gov/nchs/nhanes/index.htm.

**Figure 1:**
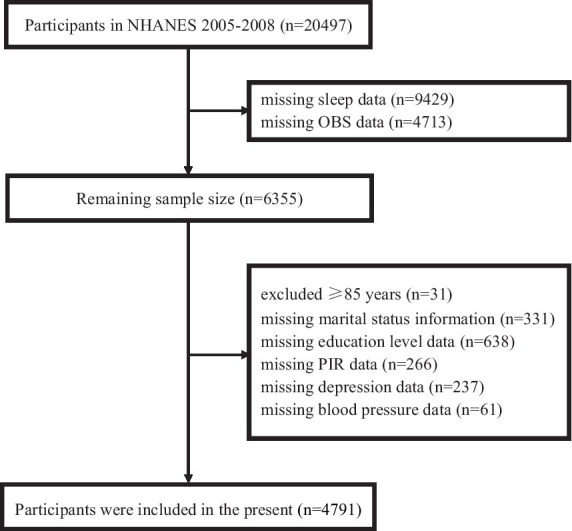
Flow chart for study participants selection.

This investigation utilized data from the NHANES cycles of 2005–2006 and 2007–2008, which included a total of 20,497 participants. To ensure the integrity and completeness of the dataset, several exclusions were made. Specifically, we excluded 9,429 participants due to missing sleep data and 4,713 participants due to missing OBS data. Additionally, 31 participants aged 85 years or older were excluded (because NHANES categorizes all individuals ≥ 85 years old as 85 years old), along with 331 participants missing marital status information, 638 participants missing education level data, 266 participants missing poverty income ratio (PIR) data, 237 participants missing depression data and 61 participants missing blood pressure data. After applying these exclusions, the final analysis included data from 4,791 participants. Further details can be found in Figure 1.

### 2.2 Assessment of sleep-related outcomes

The NHANES 2005–2008 released a comprehensive set of sleep questionnaires aligned with the Sleep Heart Health Study. In our study, we utilized the National Sleep Foundation guidelines (16) and the Healthy People 2020 (17) definition of sleep-related problems and selected the following self-reported outcomes related to sleep quality:

Sleep duration: categorized as sleep deprivation (<6 h/night), normal (7–8 h/night), or excessive (≥9 h/night) (16);

Sleep onset latency: categorized as normal (6–30 min/night), prolonged (> 30 min/night), or short (≤ 5 min/night) (18);

OSA: considered to be OSA when any of the following conditions are met: physician-diagnosed sleep apnea; snoring ≥ 3 nights per week; or snoring, gasping or stopping breathing ≥ 3 nights per week; or even though sleeping ≥ 7 h per night on weekdays and on work nights, but still feel very sleepy during the day 16–30 times per month (17);

Sleep problems: sleep problems were considered frequent if participants responded “yes,” “often” or self-reported ≥ 5 times per month in response to any of the following questions from the NHANES Sleep Questionnaire. “Have you ever told a doctor or other health professional that you have trouble sleeping?,” “In the past month, how often did you have trouble falling asleep?,” “In the past month, how often did you wake up during the night and had trouble getting back to sleep?” or “In the past month, how often did you wake up too early in the morning and were unable to get back to sleep?” (17);

Day sleepiness: Day sleepiness was considered frequent if participants answered “yes,” “often” or self-reported ≥ 5 times per month in response to any of the following questions from the NHANES Sleep Questionnaire. “In the past month, how often did you feel unrested during the day, no matter how many hours of sleep you had?” or “In the past month, how often did you feel excessively or overly sleepy during the day?” (17).

### 2.3 Assessment of OBS

OBS was calculated based on 16 dietary factors and 4 lifestyle factors that influence oxidative processes (19). The 16 dietary factors include dietary fiber, carotene, riboflavin, niacin, vitamin B6, total folate, vitamin B12, vitamin C, vitamin E, calcium, magnesium, zinc, copper, selenium, total fat and iron. The 4 lifestyle factors consist of physical activity, alcoholic consumption, body mass index (BMI) and cotinine. Among these factors, total fat, iron, BMI, alcohol consumption and smoking were classified as pro-oxidants while the remaining factors were classified as antioxidants. Variables were categorically scored from 0 to 2 based on the sex-specific tertiles of each component, and the point assignment for antioxidants and pro-oxidants was inverse (19). The scoring method for each component is detailed in Supplementary Table 1. Both antioxidants and pro-oxidants were rated on a scale from 0 to 2 points. Antioxidants received scores of 0, 1 and 2 in ascending order, while pro-oxidants were scored 0, 1 and 2 in descending order. The total OBS is the sum of the scores of the 20 constituents, with a possible range from 0 to 40 score (20). A higher score indicates a greater proportion of antioxidant exposure relative to pro-oxidant exposure.

In NHANES, participants’ dietary data were collected through two 24-h dietary recall interviews (24HR). The first 24HR was collected in the Mobile Examination Center, and the second was collected by telephone 3 to 10 days later. The dietary intake of OBS in this study was calculated as the mean of these two recalls.

The lifestyle components of OBS include alcoholic consumption, smoking, BMI and physical activity. Serum cotinine, as a major nicotine metabolite and its long half-life, has been used to assess active smoking and environmental tobacco exposure, serum cotinine levels were used to assess participants’ smoking status. Detailed descriptions of serum cotinine measurement are available on the NHANES website. BMI was from NHANES body measures collected by trained health technicians. Alcohol consumption was collected from the Alcohol Use Questionnaire, the average amount (drinks) of alcoholic beverages on those days when alcohol was consumed in the past 12 months was treated as sex-specific tertiles and subsequently assigned points (19). The metabolic equivalent (MET) scores, which were calculated from data collected by the Physical Activity Questionnaire (PAQ), were assigned for physical activity. MET serves to quantify the relative energy expenditure associated with various activities. Within the PAQ survey, the MET values corresponding to distinct categories of activities, encompassing vigorous work-related takes, moderate work-related takes, walking or bicycling for transportation, vigorous leisure-time physical activities and moderate leisure-time physical activities, were meticulously documented. Moreover, the quantification of physical activity was ascertained through the computation of the product derived by multiplying the MET value by the weekly frequency and duration of each specific physical activity (21).

### 2.4 Covariates

We included covariates based on previous studies (13, 14), including sex (male/female), age (years), race (Mexican American/other Hispanic/non-Hispanic white/non-Hispanic black/other race), PIR, education level (<high school/high school/> high school), marital status (married/divorced, widowed or separated/unmarried or cohabiting), hyperlipidemia (yes/no), diabetes mellitus (yes/no), hypertension (yes/no) and depression status.

According to previous literature (22–25), the diagnostic criteria for hyperlipidemia include total cholesterol ≥ 200 mg/dL, triglycerides ≥ 150 mg/dL, LDL ≥ 130 mg/dL, HDL < 40 mg/dL for men and < 50 mg/dL for women, or the use of lipid-lowering drugs (22). The diagnostic criteria for diabetes mellitus include self-report of having been diagnosed as diabetes mellitus by a physician, HbA1c ≥ 6.5%, use of insulin or antidiabetic medication, fasting glucose ≥ 7.0 mmol/L, random glucose ≥ 11.1 mmol/L, or 2 h glucose (from oral glucose tolerance test) ≥ 11.1 mmol/L (23). The diagnostic criteria for hypertension include self-report of having been diagnosed as high blood pressure by a physician, systolic blood pressure ≥ 140 mmHg or diastolic blood pressure ≥ 90 mmHg, or the use of antihypertensive medication (24). Depression status was assessed using the PHQ-9 questionnaire, with a score of ≥ 10 considered to be depressed (25).

### 2.5 Statistical analysis

In this study, all statistical tests were two-sided and differences were considered statistically significant at *p* < 0.050. We excluded all missing values and used R3.4.3 version^1^ and Empower software (X&Y solutions Inc., Boston, MA)^2^ for data analysis.

Given the complexity of the NHANES sampling design, we incorporated 2 years of sample weights in our analyses, following the guidelines provided in the official NHANES tutorial. Continuous variables were expressed as mean (SE) and categorical variables as percentage (SE). To compare differences in participants’ baseline characteristics, we used weighted linear regression for continuous variables and weighted chi-square tests for categorical variables. To estimate the linear relationship between the OBS and sleep-related problems, we constructed logistic regression models, adjusting for a range of confounders. Confounders were selected on the basis that their correlation with the outcome or effect estimate varied by more than 10% (26). Model 1 was an unadjusted model. Model 2 was adjusted for age, gender and race. Model 3 was further adjusted for blood pressure, PIR, depression, diabetes, hyperlipidemia, education and marital status, based on Model 2. To explore the nonlinear relationship between OBS and the risk of developing sleep-related problems, generalized additive models was employed. Upon identifying a nonlinear correlation, we applied segmented regression (also known as piece-wise regression) that uses a separate line segment to fit each interval. A log-likelihood ratio test was performed to compare the one-line (non-segmented) model with segmented regression model, in order to determine whether a threshold exists. Then the K value of the fitted model with the maximum likelihood was identified using a two-step recursive method, which serves as the inflection point connecting the segmented intervals (27). We also treated OBS as a continuous variable in these logistic models to perform trend tests between OBS and sleep-related problems. Finally, subgroup analyses and interaction tests were conducted to identify potential interactions across different population settings and demographics. Additionally, we explored the potential for unmeasured confounding between sleep-related outcomes and OBS by calculating *E*-values (28). The *E*-value quantifies the required magnitude of an unmeasured confounder that could negate the observed association between sleep-related outcomes and OBS.

## 3 Results

### 3.1 Baseline characteristics of participants

A total of 4,791 participants were included in this study, with a mean age of 45.23 ± 0.51 years. Among them, 2,503 (50.99%) were male, and 2,317 (49.01%) were female. Participants with insufficient sleep hours, excessive sleep onset latency, OSA, day sleepiness, sleep problems were 34.83, 15.29, 55.71, 29.84 and 40.38%, respectively. Significant differences (*p* < 0.05) in the OBS were observed across OBS quartiles with varying PIR, education levels, race, marital status, and the presence of diabetes, hypertension, hyperlipidemia, or depression. The specific characteristics of the participants are detailed in Table 1.

**Table 1:** General analysis of the study population.

Variables		Total (*n* = 4,791)	Q1(*n* = 1,165)	Q2(*n* = 1,102)	Q3(*n* = 1,321)	Q4(*n* = 1,203)	*p*-value
Age		45.23 (0.51)	45.12 (0.59)	44.84 (0.53)	44.91 (0.65)	45.96 (0.84)	0.378
Gender	Male	50.99 (0.78)	53.03 (1.48)	51.47 (1.78)	51.84 (1.16)	48.19 (1.94)	0.168
	Female	49.01 (0.78)	46.97 (1.48)	48.53 (1.78)	48.16 (1.16)	51.81 (1.94)	
Marital status	Married	62.63 (1.38)	56.60 (2.03)	58.81 (2.12)	63.54 (1.93)	69.24 (1.72)	<0.001
	Separated/widowed/divorced	14.20 (0.68)	15.70 (1.14)	15.03 (1.35)	14.54 (1.29)	12.05 (0.99)	
	Never married/living with partner	23.17 (1.25)	27.70 (1.98)	26.16 (1.69)	21.93 (1.49)	18.70 (1.57)	
Educational level	<High school	14.40 (1.02)	19.91 (1.54)	17.29 (1.71)	12.35 (1.32)	10.12 (1.13)	<0.001
	High school	22.95 (1.03)	30.67 (1.61)	25.34 (1.61)	21.29 (1.75)	16.98 (1.44)	
	> High school	62.64 (1.80)	49.42 (1.80)	57.36 (2.59)	66.36 (2.22)	72.90 (1.99)	
Race	Mexican American	6.41 (0.63)	6.63 (0.89)	7.07 (0.77)	6.17 (0.76)	5.99 (0.76)	<0.001
	Non-Hispanic White	76.43 (1.99)	70.49 (2.79)	72.42 (2.68)	78.28 (2.15)	82.15 (1.92)	
	Non-Hispanic Black	8.62 (1.20)	14.76 (1.88)	10.19 (1.67)	7.44 (1.28)	3.97 (0.63)	
	Other Hispanic	3.61 (0.55)	3.86 (0.95)	4.41 (0.76)	4.04 (0.69)	2.35 (0.48)	
	Other Race	4.92 (0.48)	4.25 (0.83)	5.90 (1.08)	4.08 (0.64)	5.55 (1.19)	
PIR		3.29 (0.06)	2.85 (0.07)	3.15 (0.08)	3.40 (0.07)	3.64 (0.07)	<0.001
Hyperlipidemia	No	29.53 (1.06)	24.31 (2.19)	28.69 (1.91)	30.11 (1.34)	33.52 (1.24)	<0.001
	Yes	70.47 (1.06)	75.69 (2.19)	71.31 (1.91)	69.89 (1.34)	66.48 (1.24)	
Hypertension	No	65.95 (0.93)	60.77 (1.87)	64.16 (1.62)	68.32 (1.77)	68.78 (1.71)	<0.003
	Yes	34.05 (0.93)	39.23 (1.87)	35.84 (1.62)	31.68 (1.77)	31.22 (1.71)	
Diabetes	No	89.93 (0.60)	87.10 (1.48)	89.72 (1.19)	90.02 (1.02)	92.14 (0.66)	<0.009
	Yes	10.07 (0.60)	12.90 (1.48)	10.28 (1.19)	9.98 (1.02)	7.86 (0.66)	
Depression	No	94.60 (0.37)	90.63 (0.95)	93.71 (0.99)	95.18 (0.68)	97.70 (0.43)	<0.001
	Yes	5.40 (0.37)	9.37 (0.95)	6.29 (0.99)	4.82 (0.68)	2.30 (0.43)	
Sleep duration	Sufficient	65.17 (1.05)	57.91 (1.60)	63.18 (2.03)	64.90 (1.71)	72.51 (1.93)	<0.001
	Short	34.83 (1.05)	42.09 (1.60)	36.82 (2.03)	35.10 (1.71)	27.49 (1.93)	
Sleep onset latency	Suitable	84.71 (0.68)	78.84 (1.43)	84.31 (1.17)	86.54 (0.95)	87.54 (1.30)	<0.001
	Excessive	15.29 (0.68)	21.16 (1.43)	15.69 (1.17)	13.46 (0.95)	12.46 (1.30)	
OSA	No	44.29 (1.19)	39.06 (2.56)	40.33 (2.04)	45.21 (1.47)	50.40 (1.54)	<0.001
	Yes	55.71 (1.19)	60.94 (2.56)	59.67 (2.04)	54.79 (1.47)	49.60 (1.54)	
Sleep problems	No	59.62 (0.78)	56.86 (1.44)	60.30 (1.49)	60.34 (1.56)	60.43 (1.18)	0.227
	Yes	40.38 (0.78)	43.14 (1.44)	39.70 (1.49)	39.66 (1.56)	39.57 (1.18)	
Day sleepiness	No	70.16 (0.84)	69.43 (1.61)	67.62 (1.67)	70.30 (1.63)	72.58 (1.80)	0.219
	Yes	29.84 (0.84)	30.57 (1.61)	32.38 (1.67)	29.70 (1.63)	27.42 (1.80)	

Continuous variables were expressed as mean (SE) and categorical variables as percentage (SE). OBS: Oxidative Balance Score; PIR: Ratio of family income to poverty. Q1: OBS<15 score; Q2: 15 ≤ OBS<20 score; Q3: 20 ≤ OBS<25 score; Q4: 25 ≤ OBS score.

### 3.2 Relationship between OBS and sleep-related outcomes

#### 3.2.1 Logistic analysis of OBS and sleep-related outcomes

Our findings indicate that participants with low OBS were at a greater risk of experiencing insufficient sleep hours and developing OSA. The association between OBS and insufficient sleep hours was significantly correlated in the original model (OR = 0.96, 95% CI 0.95–0.97, *p* < 0.001) and the minimum-adjusted model (OR = 0.97, 95% CI 0.95–0.98, *p* < 0.001). In the fully adjusted model, this association remained stable (OR = 0.98, 95% CI 0.96–0.99, *p* = 0.004). Similarly, a significant association was observed between OBS and the risk of developing OSA. In the original model (OR = 0.97, 95% CI 0.96–0.98, *p* < 0.001) and in the minimum-adjusted model (OR = 0.97, 95% CI 0.95–0.98, *p* < 0.001). This relationship also remained consistent in the fully adjusted model (OR = 0.97, 95% CI 0.96–0.99, *p* = 0.003). Further details are presented in Table 2.

**Table 2:** Logistic and trend analysis of OBS and sleep-related outcomes.

	Model 1	Model 2	Model 3
	OR(95%CI)*P*	OR(95%CI)*P*	OR(95%CI)*P*
Sleep duration	Short vs. sufficient		
OBS	0.96 (0.95, 0.97) < 0.001	0.97 (0.95, 0.98) < 0.001	0.98 (0.96, 0.99)0.004
Q1	Reference	Reference	Reference
Q2	0.80 (0.64, 1.01)0.072	0.82 (0.66, 1.03)0.105	0.87 (0.70, 1.09)0.255
Q3	0.74 (0.61, 0.91)0.007	0.79 (0.65, 0.97)0.036	0.87 (0.70, 1.09)0.249
Q4	0.52 (0.40, 0.67) < 0.001	0.57 (0.45, 0.74) < 0.001	0.66 (0.50, 0.88)0.014
*P* for trend	<0.001	<0.001	0.014
Sleep onset latency	Excessive vs. Suitable		
OBS	0.96 (0.94, 0.97) < 0.001	0.96 (0.94, 0.98) < 0.001	0.98 (0.96, 1.00)0.138
Q1	Reference	Reference	Reference
Q2	0.69 (0.57, 0.84) < 0.001	0.71 (0.58, 0.85)0.002	0.80 (0.63, 1.01)0.055
Q3	0.58 (0.46, 0.73) < 0.001	0.61 (0.47, 0.77) < 0.001	0.77 (0.57, 1.04)0.081
Q4	0.53 (0.39, 0.72) < 0.001	0.57 (0.41, 0.79)0.003	0.82 (0.55, 1.22)0.296
*P* for trend	<0.001	0.002	0.270
OSA	Yes vs. No		
OBS	0.97 (0.96, 0.98) < 0.001	0.97 (0.95, 0.98) < 0.001	0.97 (0.96, 0.99)0.003
Q1	Reference	Reference	Reference
Q2	0.95 (0.76, 1.18)0.637	0.96 (0.77, 1.21)0.736	1.01 (0.82, 1.25)0.899
Q3	0.78 (0.63, 0.96)0.024	0.77 (0.62, 0.97)0.038	0.83 (0.66, 1.05)0.148
Q4	0.63 (0.50, 0.80) < 0.001	0.63 (0.48, 0.82)0.002	0.70 (0.54, 0.92)0.024
*P* for trend	<0.001	<0.001	0.009
Sleep problems	Yes vs. No		
OBS	0.99 (0.98, 1.00)0.014	0.98 (0.98, 0.99) < 0.001	1.00 (0.98, 1.01)0.295
Q1	Reference	Reference	Reference
Q2	0.87 (0.73, 1.03)0.107	0.86 (0.72, 1.02)0.092	0.92 (0.75, 1.12)0.371
Q3	0.87 (0.74, 1.02)0.079	0.84 (0.72, 0.98)0.040	0.94 (0.77, 1.14)0.496
Q4	0.86 (0.74, 1.00)0.055	0.81 (0.70, 0.93)0.008	0.97 (0.79, 1.18)0.734
*P* for trend	0.069	0.006	0.842
Day sleepiness	Yes vs. No		
OBS	0.99 (0.98, 1.00)0.048	0.99 (0.97, 1.00)0.024	1.00 (0.98, 1.01)0.722
Q1	Reference	Reference	Reference
Q2	1.09 (0.88, 1.35)0.453	1.07 (0.86, 1.33)0.534	1.19 (0.91, 1.55)0.190
Q3	0.96 (0.76, 1.21)0.724	0.93 (0.74, 1.17)0.548	1.06 (0.80, 1.41)0.644
Q4	0.86 (0.70, 1.05)0.143	0.82 (0.66, 1.01)0.072	1.02 (0.78, 1.32)0.899
*P* for trend	0.063	0.031	0.790

Model 2 adjusted for gender, age, and race; Model 3 adjusted for education level, marital status, PIR, and the presence of hypertension, diabetes, hyperlipidemia, and depression on top of the Model 2.

#### 3.2.2 Trend analysis of OBS and sleep-related outcomes

To further perform sensitivity analyses, we transformed the continuous variable OBS into a categorical variable. Regarding the risk of developing OSA, the highest quartile demonstrated a statistically significant 30% reduction in risk compared to the lowest quartile (OR = 0.70, 95% CI 0.54–0.92, *p* = 0.024). In contrast, the Q2 group showed 1% reduction in risk and the Q3 group showed 17% reduction in the risk of developing OSA compared to the lowest quartile. However, these associations were not statistically significant. Further details are presented in Table 2.

#### 3.2.3 The analyses of non-linear relationship

**Figure 2:**
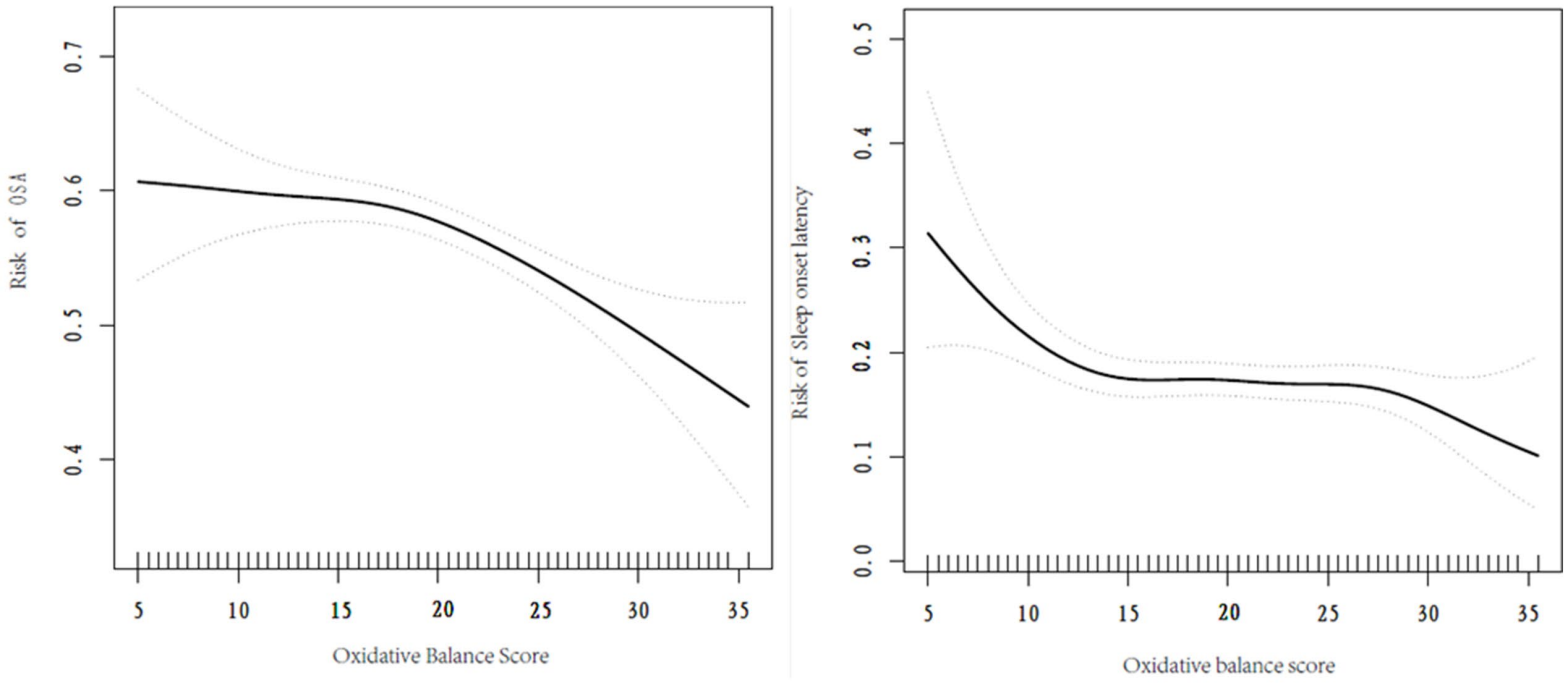
Smoothed curve fitting plot.

A nonlinear relationship was observed between OBS and the risk of developing OSA as well as excessive sleep onset latency. Utilizing a two-part linear regression model, we calculated that the inflection point between OBS and the risk of developing OSA was 17.5 score, when OBS < 17.5 score, the effect value was 1.01 (95% CI 0.98–1.04, *p* = 0.443), when OBS > 17.5, the effect value was 0.96 (95% CI 0.95–0.98, *p* < 0.001). 10.5 score was the inflection point between OBS and the risk of excessive sleep onset latency, when OBS < 10.5 score, the effect value was 0.84 (95% CI 0.73–0.96, *p* = 0.009), when OBS > 10.5 score, the effect value was 0.99 (95% CI 0.97–1.00, *p* = 0.111). Further details can be found in Table 3 and Figure 2.

**Table 3:** Analysis of non-linear relationships between OBS and sleep-related outcomes.

OBS	OSA	Sleep Onset latency
	OR (95%CI)*P*	OR (95%CI)*P*
Model I		
OR	0.98 (0.97, 0.99)< 0.001	0.98 (0.97, 0.99)<0.005
Model II		
Breakpoint (K)	17.5	10.5
<K OR 1	1.01 (0.98, 1.04)0.443	0.84 (0.73, 0.96)0.009
> K OR 2	0.96 (0.95, 0.98)< 0.001	0.99 (0.97, 1.00)0.111
OR2/OR1	0.95 (0.92, 0.99)0.013	1.18 (1.03, 1.36)0.019
Log-likelihood ratio test	0.013	0.021

Adjustment variables gender, age, race, education level, marital status, PIR, and presence of hypertension, diabetes, hyperlipidemia, and depression.

#### 3.2.4 Subgroup analysis and *E*-value

The results of the subgroup analyses are presented in Table 4. After adjusting for confounders, the negative associations between OBS and insufficient sleep hours as well as the risk of developing OSA, remained stable. Furthermore, the interaction test revealed that the correlation between OBS and sleep-related problems did not significantly differ across various strata (all interaction *p* > 0.05). This indicates that the relationship between OBS and sleep-related problems is consistent and unaffected by age, gender, race, etc. For more detailed information, please refer to Table 4. Additionally, we calculated E values to evaluate the sensitivity of our findings to potential unmeasured confounding factors. As demonstrated in Table 5, the E values suggested that our results are robust, provided that there are no unmeasured confounders with odds ratio exceed the E values associated with sleep-related outcomes.

**Table 4:** Subgroup analysis.

	OSA		Sleep onset latency		Sleep duration	
	OR(95%CI) interaction		OR(95%CI) interaction		OR(95%CI) interaction	
Age		0.283		0.161		0.771
<60	0.97 (0.96, 0.99)		0.98 (0.96, 1.00)		0.97 (0.96, 0.99)	
≥60	0.99 (0.97, 1.01)		1.00 (0.98, 1.02)		0.97 (0.95, 0.99)	
Gender		0.282		0.055		0.092
Male	0.98 (0.97, 1.00)		0.99 (0.98, 1.01)		0.98 (0.97, 1.00)	
Female	0.97 (0.96, 0.99)		0.97 (0.95, 0.99)		0.96 (0.95, 0.98)	
Race		0.548		0.447		0.323
Mexican American	0.99 (0.97, 1.02)		0.99 (0.96, 1.02)		0.98 (0.97, 1.00)	
Other Hispanic	0.99 (0.95, 1.03)		0.97 (0.93, 1.01)		0.98 (0.95, 1.01)	
Non-Hispanic White	0.97 (0.96, 0.99)		0.98 (0.96, 1.00)		0.97 (0.95, 0.98)	
Non-Hispanic Black	0.99 (0.97, 1.01)		1.00 (0.98, 1.03)		0.98 (0.96, 1.01)	
Other race	0.98 (0.93, 1.03)		0.97 (0.90, 1.04)		0.96 (0.90, 1.01)	
Educational level		0.156		0.313		0.360
<High school	0.98 (0.96, 1.01)		0.98 (0.96, 1.00)		0.97 (0.94, 1.00)	
High school	0.99 (0.97, 1.01)		1.00 (0.97, 1.03)		0.98 (0.96, 1.01)	
> High school	0.97 (0.96, 0.98)		0.98 (0.96, 1.00)		0.97 (0.96, 0.98)	

**Table 5:** *E*-values in the fully adjusted model.

*E*-value for OR estimate	Variable	Level	OR (95% CI)
1.76	Sleep duration	Q4 vs. Q1	0.66 (0.50, 0.88)
1.68	OSA	Q4 vs. Q1	0.70 (0.54, 0.92)

## 4 Discussion

In this study, we identified a negative association between OBS and the risk of insufficient sleep hours, indicating that participants with lower OBS are at a higher risk of experiencing insufficient sleep hours. We observed a nonlinear relationship between OBS and both the risk of developing OSA and having excessive sleep onset latency, with differing characteristics on either side of the turning point. Specifically, the association between OBS and the risk of developing OSA was not significant on the left side of the turning point. However, it became significant on the right side, where higher OBS corresponded to a reduced risk of developing OSA. Conversely, the relationship between OBS and the risk of having excessive sleep onset latency was significant on the left side of the turning point (indicating that higher OBS is associated with a lower risk), but this relationship lost significance on the right side. Meanwhile, our subgroup analyses and interaction tests further confirmed that these relationships remained stable across various population settings. Our findings demonstrate the impact of oxidative homeostasis on sleep and provide evidence supporting increased intake of antioxidant-rich foods and an antioxidant lifestyle.

Notably, our study found that OBS was negatively correlated with the risk of insufficient sleep (OR = 0.98, *p* < 0.05). This aligns with findings from Lei X and colleagues (13), who identified a significant association between OBS and sleep duration (MD = 0.26, p < 0.05) using a different research design. Additionally, research by Zhang Q and colleagues (29) revealed a positive dose–response relationship between OBS and sleep duration, indicating that higher OBS is associated with longer sleep duration (*β* = 0.009, *p* < 0.05). Despite methodological differences among the three studies, they consistently confirm the robustness of the negative association between OBS and the risk of insufficient sleep duration. Moreover, our study reveals for the first time a nonlinear relationship between OBS and the risk of developing OSA and excessive sleep onset latency, our results are similar to the result of Xiong B and colleagues (30) who found a nonlinear relationship between the composite dietary antioxidant index (CDAI) and OSA. CDAI is a composite index like OBS, and there are many commonalities in the components contained. Our study explored the relationship between OBS and sleep-related problems and the results were generally reasonable. Although the underlying biological mechanisms linking OBS and sleep-related problems are currently unknown, we propose several explanations based on existing knowledge:

First, a good diet and lifestyle influence the oxidative stress of the organism. Among lifestyle factors, for example, smoking inhibits the body’s antioxidant defense mechanisms and promotes the production of endogenous free radicals (31). Studies have shown that smokers have significantly lower serum enzymes with antioxidant effects (superoxide dismutase (32)), plasma antioxidant molecules [melatonin and *β*-carotene (33, 34)], and significantly higher levels of oxidative stress markers [plasma malondialdehyde (32)]. Oxidative stress, in turn, impacts sleep quality through neurobiological mechanisms associated with oxidative processes. For example, brain tissue is particularly vulnerable to oxidative stress due to its high metabolic demand and low levels of endogenous antioxidants. The numerous reactive oxygen radicals produced during oxidative stress contributes to impaired sleep quality through a variety of mechanisms, including damage to endothelial cells and reduction of neurotransmitter concentrations (35). The OBS is calculated based on the level of oxidative exposure of an individual based on 16 dietary and 4 lifestyle factors that influence oxidative processes, with higher scores reflecting better antioxidant exposure. Therefore, it is reasonable to conclude that individuals with higher OBS are at lower risk of developing sleep-related problems.

Second, a balanced diet and healthy lifestyle are crucial in reducing systemic inflammation. Inflammatory factors involved in the immune response and inflammatory reaction, and it plays a significant role in the neuro-endocrine-immune network, participating in the regulation of sleep–wake rhythms. When inflammatory factors TNF-*α*, IL-1β, and IL-6 are moderately increased, the body can compensate for sleep by increasing slow-wave sleep (36). However, when the levels of these inflammatory markers are significantly elevated, excessive inflammatory factors not only do not promote sleep, but also induce the production of neurotoxic substances in the central nervous system to aggravate insomnia (37, 38). Therefore, managing the levels of inflammatory factors is essential for maintaining sleep quality. Research indicates that a nutritious diet and regular physical activity can reduce pro-inflammatory factors (39, 40), inversely smoking and alcohol consumption can increase their production (41, 42). Previous researches reported that dietary zinc and selenium had both properties of antioxidant and pro-oxidant, which could cause oxidative stress and lose antioxidant capacity when they were out of certain range (43, 44). These findings offer insight into the paradox between OBS and OSA and prolonged sleep onset from various perspectives. More investigations are needed in the future to understand the biological mechanisms between these relationships.

Our study has some strengths. Firstly, the research data were sourced from the NHANES database, which is known for its large scale and national representativeness. This ensuring the reliability of our findings. Secondly, we employed OBS, a comprehensive index that considers both dietary and lifestyle factors influencing oxidative stress. However, our study also has limitations. Primarily, the cross-sectional design meant that all measurements were taken only at baseline, without dynamic follow-up, necessitating further prospective studies to establish causality. Additionally, sleep-related problems were assessed based on self-reports of participants during interviews, lacking objective measures, which may introduce information bias. Lastly, although numerous covariates were adjusted, the influence of other confounding factors cannot be entirely excluded. We used the *E*-value sensitivity analysis to quantify the potential implications of unmeasured confounders and found that an unmeasured confounder was unlikely to explain the entirety of the related-sleep outcomes.

## 5 Conclusion

The findings of the present study reveal an inverse linear relationship between OBS and the risk of insufficient sleep hours, alongside a nonlinear relationship between OBS and the risks of developing OSA and excessive sleep onset latency. These results suggest that optimizing the body’s oxidative balance may enhance sleep quality in the population.

## Data Availability

Publicly available datasets were analyzed in this study. This data can be found here: https://www.cdc.gov/nchs/nhanes/.

## Footnotes

^1^ http://www.R-project.org

^2^ www.empowerstats.com

## Supplementary material

The Supplementary material for this article can befound online at: https://www.frontiersin.org/articles/10.3389/fnut.2025.1571971/full#supplementary-material
