# Supplementary material for: Relationship between oxidative balance score and risk of sleep-related problems
Source: Front Nutr. 2025 Apr 28;12:1571971. doi: 10.3389/fnut.2025.1571971 (PMC12066617; doi:10.3389/fnut.2025.1571971)
Supplement: Supplementary file 1 [file Table_1.docx]

Supplemental **Table 1 Oxidative balance score (OBS) items and score assignment**

| OBS components | Property | Male | | | Female | | |
| --- | --- | --- | --- | --- | --- | --- | --- |
|  |  | 0 | 1 | 2 | 0 | 1 | 2 |
| Dietary OBS components | |  |  |  |  |  |  |
| Dietary fiber (g/d) | A | <13.55 | 13.55-20.40 | ≥20.40 | <11.85 | 11.85-17.95 | ≥17.95 |
| Carotene (RE/d) | A | <595.50 | 595.50-1843.00 | ≥1843.00 | <660.50 | 660.50-2276.50 | ≥2276.50 |
| Riboflavin (mg/d) | A | <1.89 | 1.89-2.71 | ≥2.71 | <1.50 | 1.50-2.12 | ≥2.12 |
| Niacin (mg/d) | A | <25.13 | 25.13-34.51 | ≥34.51 | <17.53 | 17.53-24.29 | ≥24.29 |
| Vitamin B6 (mg/d) | A | <1.86 | 1.86-2.73 | ≥2.73 | <1.36 | 1.36-1.99 | ≥1.99 |
| Total folate (mcg/d) | A | <343.00 | 343.00-500.50 | ≥500.50 | <267.50 | 267.50-394.50 | ≥394.50 |
| Vitamin B12 (mcg/d) | A | <3.87 | 3.87-6.51 | ≥6.51 | <2.67 | 2.67-4.58 | ≥4.58 |
| Vitamin C (mg/d) | A | <37.85 | 37.085-96.35 | ≥96.35 | <38.60 | 38.60-86.15 | ≥86.15 |
| Vitamin E (ATE) (mg/d) | A | <6.58 | 6.58-10.16 | ≥10.16 | <5.74 | 5.74-8.77 | ≥8.77 |
| Calcium (mg/d) | A | <812.50 | 812.50-1230.00 | ≥1230.00 | <670.00 | 670.00-990.00 | ≥990.00 |
| Magnesium (mg/d) | A | <276.50 | 276.500-377.00 | ≥377.00 | <226.50 | 226.50-311.00 | ≥311.00 |
| Zinc (mg/d) | A | <10.40 | 10.40-14.88 | ≥14.88 | <7.59 | 7.59-10.66 | ≥10.66 |
| Copper (mg/d) | A | <1.07 | 1.07-1.51 | ≥1.51 | <0.91 | 0.91-1.27 | ≥1.27 |
| Selenium (mcg/d) | A | <109.70 | 109.70-150.55 | ≥150.55 | <77.45 | 77.45-108.35 | ≥108.35 |
| Total fat (g/d) | P | ≥105.93 | 74.87-105.93 | <74.87 | ≥79.69 | 56.24-79.69 | <56.24 |
| Iron (mg/d) | P | ≥18.21 | 12.84-18.21 | <12.84 | ≥14.04 | 9.91-14.04 | <9.91 |
| Lifestyle OBS components |  |  |  |  |  |  |  |
| Physical activity (MET-minute/week) | A | <1920 | 1920-6060 | ≥6060 | <1080 | 1080-3120 | ≥3120 |
| Acoholic drinks at past 12 months (drink/d) | P | >3 | 2-3 | ≤2 | >2 | 1-2 | ≤1 |
| Body mass index (kg/m2) | P | ≥30.05 | 25.71-30.05 | <25.71 | ≥29.90 | 24.00-29.90 | <24.00 |
| Cotinine (ng/mL) | P | ≥3.070 | 0.022-3.070 | <0.022 | ≥0.094 | 0.011-0.094 | <0.011 |

OBS: oxidative balance score; A: antioxidant; P: pro-oxidant; RE: retinol equivalent; ATE: alpha-tocopherol equivalent; MET: metabolic equivalent.
